# *Mycobacterium gordonae* in Patient with Facial Ulcers, Nosebleeds, and Positive T-SPOT.*TB* Test, China

**DOI:** 10.3201/eid2307.162033

**Published:** 2017-07

**Authors:** Yanqing Chen, Juan Jiang, Haiqin Jiang, Jun Chen, Xiaopo Wang, Weida Liu, Zhiming Chen, Ying Shi, Wenyue Zhang, Hongsheng Wang

**Affiliations:** Institute of Dermatology, Chinese Academy of Medical Sciences and Peking Union Medical College, Nanjing, China (Y. Chen, J. Jiang, H. Jiang, X. Wang, W. Liu, Z. Chen, Y. Shi, W. Zhang, H. Wang);; Jiangsu Key Laboratory of Molecular Biology for Skin Diseases and STIs, Nanjing, (Y. Chen, J. Jiang, H. Jiang, X. Wang, W. Liu, Z. Chen, Y. Shi, W. Zhang, H. Wang);; Jiangsu Provincial Cancer Hospital, Nanjing (J. Chen)

**Keywords:** nontuberculous mycobacteria, interferon-gamma release tests, skin diseases, nose diseases, ulcer, nosebleed, T-SPOT.TB, tuberculosis and other mycobacteria, China, *Mycobacterium gordonae*

## Abstract

*Mycobacterium gordonae* is often regarded as a weak pathogen that only occasionally causes overt disease. We report a case of *M. gordonae* infection in the facial skin, nasal mucosa, and paranasal sinus in an immunocompetent patient and review previous cases. The T-SPOT.*TB* test might be useful in diagnosing such cases.

Nontuberculous mycobacteria are increasingly more involved in causing human infections. *Mycobacterium gordonae*, a type of slow-growing nontuberculous mycobacterium, is generally regarded as a weak pathogen, although it has caused some disease in humans (*1**–**3*). We describe a case of *M. gordonae* infection in the facial skin, nasal mucosa, and paranasal sinus of an immunocompetent patient in China.

In March 2016, a 60-year-old woman with no history of tuberculosis or immunosuppression sought treatment for a 2-year history of asymptomatic facial ulcers and intermittent nosebleeds. The primary lesion was a small erythema on the left cheek that, without incurring trauma, developed into an ulcer. The condition gradually worsened. One year before visiting our hospital, she received a diagnosis of lupus erythematosus; she had also been referred to otorhinolaryngology, where she was treated unsuccessfully with nasal endoscopic surgery. The skin lesions were erythematous, covered with yellow crusts, and on both sides of the patient’s face (Figure, panel A). A nasal examination revealed small scabs in the nasal vestibule. Lymph nodes were not palpable.

**Figure:**
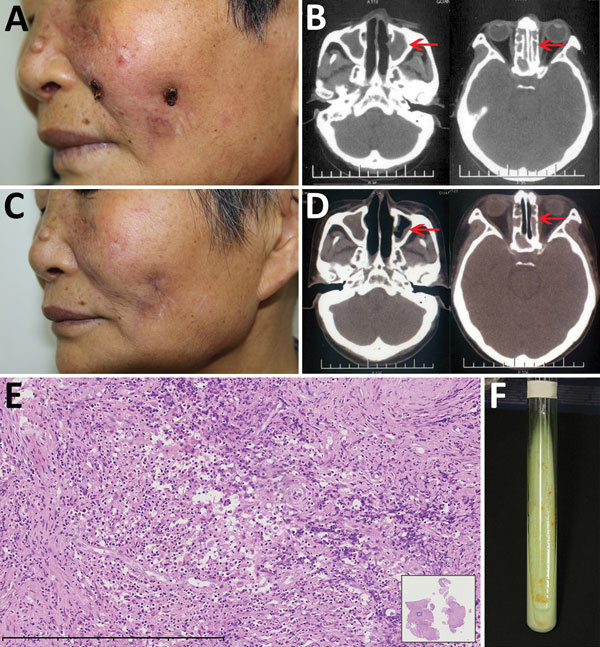
*Mycobacterium gordonae* infection in a 60-year-old immunocompetent woman, China. A) Facial lesions before treatment. Ulcers were erythematous and covered with yellow crusts. B) Computed tomography images before treatment show heterogeneous hypersignal in the ethmoid and left maxillary sinus (arrows). C) Facial lesions after treatment. Atrophic scars are seen at sites of previous lesions. D) Computed tomography images after treatment show recovery of the ethmoid sinus and left maxillary sinus (arrows). E) Hematoxylin and eosin stain of nasal mucosa showing the infiltration of a large number of lymphocytes, a few histiocytes, and plasma cells. Scale bar corresponds to 400 µm. Inset shows the nasal mucosa sample (original magnification ×20). F) Tissue culture 3 weeks after incubation shows yolk-yellow bacteria growing in Löwenstein–Jensen medium. A color version of this figure is available online (https://wwwnc.cdc.gov/EID/article/23/7/16-2033-F1.htm).

Laboratory test results indicated the patient was negative for autoantibodies and HIV. A computed tomography (CT) scan of the paranasal sinuses showed ethmoid and maxillary sinusitis (Figure, panel B). Histologic examination indicated that a large number of lymphocytes, a few histiocytes, and plasma cells had infiltrated the lesion (Figure, panel E). Periodic acid-Schiff and acid-fast stains were negative for bacteria, and tissue culture was negative for fungus. Mycobacterial infection was suspected, but because mycobacteria are slow-growing, another test was needed to inform clinical practice. An alternative diagnostic test, the T-SPOT.*TB* (Oxford Immunotec Ltd., Oxford, England), an interferon-γ release assay, was performed and showed positive results (tuberculosis [TB] antigen – Nil = 20; >6 was positive). After 3 weeks of culture at 32°C in Löwenstein–Jensen medium, smooth, creamy, yolk-yellow bacteria were observed (Figure, panel F). Ziehl–Neelsen staining indicated they were acid-fast bacilli. Sequence analysis of 16S rRNA revealed a 99% similarity with *M. gordonae* strain Y27, and the *hsp65* gene showed a 100% similarity with *M. gordonae* strain FJ09081. Further sequence analysis showed 16S rRNA was 99% homologous and *hsp65* 96% homologous with *M. gordonae* ATCC 14470. The diagnosis of *M. gordonae* infection was made.

After diagnosis, the patient was empirically treated with clarithromycin (500 mg/d) and moxifloxacin (400 mg/d). At the 4-month follow-up, no new nosebleeds or skin lesions were reported, and skin examination revealed scattered atrophic scars on the face (Figure, panel C). CT scans showed substantial improvement of the ethmoid and maxillary sinuses (Figure, panel D); T-SPOT.*TB* results were negative (TB antigen – Nil = 0). Treatment continued for 2 more months.

A drug sensitivity test showed that the isolate from the patient was sensitive to clarithromycin, ethambutol, and moxifloxacin but less sensitive to rifampin, rifabutin, and isoniazid (*4*). No adverse drug reactions or recurrences were noted for up to 3 months after treatment was completed.

*M. gordonae* is found widely throughout the environment. Infections caused by *M. gordonae* usually occur in the lungs (and only occasionally in other organs) of immunocompromised patients. A cutaneous infection with *M. gordonae* is unusual and a paranasal sinus infection even rarer.

Seven cases of cutaneous infection with *M. gordonae* have been reported; all were in women 38–80 years of age. The infection can affect persons who have not experienced trauma or been exposed to immunosuppressants. The most common lesions caused by *M. gordonae* are nodules that slowly enlarge and become ulcerated over several months. Lesions usually are located on the face, at distal extremities, or at sites of previous trauma (*1**,**5*–*8*). In this case, the infection caused lesions throughout the facial skin, nasal mucosa, and paranasal sinus, probably because the delay in accurate diagnosis allowed for wide dissemination of the pathogen.

Common laboratory methods for diagnosis of nontuberculous mycobacterial infection include histopathologic stainings, tissue culture, PCR, and gene sequencing. Tissue culture and sequencing usually provide the most reliable evidence for diagnosis; however, tissue culture has a low sensitivity and is time-consuming, making early diagnosis difficult.

In this case, the initial patient sample was positive by T-SPOT.*TB*, and after mycobacterium-specific treatment and clinical improvement, the convalescent-phase patient sample was negative by T-SPOT.*TB*. These results were highly suggestive that the patient had a mycobacterial infection and that the infection was adequately treated.

Although it is generally believed that a positive T-SPOT.*TB* result means the patient has an *M. tuberculosis* infection, positive results have been reported for infections caused by nontuberculous mycobacteria (*2**,**9*). The T-SPOT.*TB* assay uses the *M. tuberculosis* antigens ESAT-6 and CFP-10. The *ESAT-6* and *CFP-10* genes are located within the *M. tuberculosis* region of difference 1 (RD1), a DNA sequence that is also present in *M. marinum*, *M. kansasii*, *M. szulgai*, and *M. gordonae* (*10*). Because these genes are present in other mycobacterial genomes, the T-SPOT.*TB* assay might be useful for diagnosing infections with multiple RD1-possessing mycobacteria. However, further studies are needed to confirm its diagnostic value.
